# Precise healing of oral and maxillofacial wounds: tissue engineering strategies and their associated mechanisms

**DOI:** 10.3389/fbioe.2024.1375784

**Published:** 2024-04-18

**Authors:** Qingtong Zhao, Changyun Leng, Manting Lau, Kawai Choi, Ruimin Wang, Yuyu Zeng, Taiying Chen, Canyu Zhang, Zejian Li

**Affiliations:** ^1^ Hospital of Stomatology, The First Affiliated Hospital of Jinan University, Guangzhou, China; ^2^ Department of Stomatology, The Sixth Affiliated Hospital of Jinan University, Dongguan, China; ^3^ School of stomatology, Jinan University, Guangzhou, China; ^4^ Department of Stomatology, Baoan Central Hospital of Shenzhen, Shenzhen, China

**Keywords:** wound healing, tissue engineering, hydrogel, nanofibers, tissue repair

## Abstract

Precise healing of wounds in the oral and maxillofacial regions is usually achieved by targeting the entire healing process. The rich blood circulation in the oral and maxillofacial regions promotes the rapid healing of wounds through the action of various growth factors. Correspondingly, their tissue engineering can aid in preventing wound infections, accelerate angiogenesis, and enhance the proliferation and migration of tissue cells during wound healing. Recent years, have witnessed an increase in the number of researchers focusing on tissue engineering, particularly for precise wound healing. In this context, hydrogels, which possess a soft viscoelastic nature and demonstrate exceptional biocompatibility and biodegradability, have emerged as the current research hotspot. Additionally, nanofibers, films, and foam sponges have been explored as some of the most viable materials for wound healing, with noted advantages and drawbacks. Accordingly, future research is highly likely to explore the application of these materials harboring enhanced mechanical properties, reduced susceptibility to external mechanical disturbances, and commendable water absorption and non-expansion attributes, for superior wound healing.

## 1 Introduction

**FIGURE 1:**
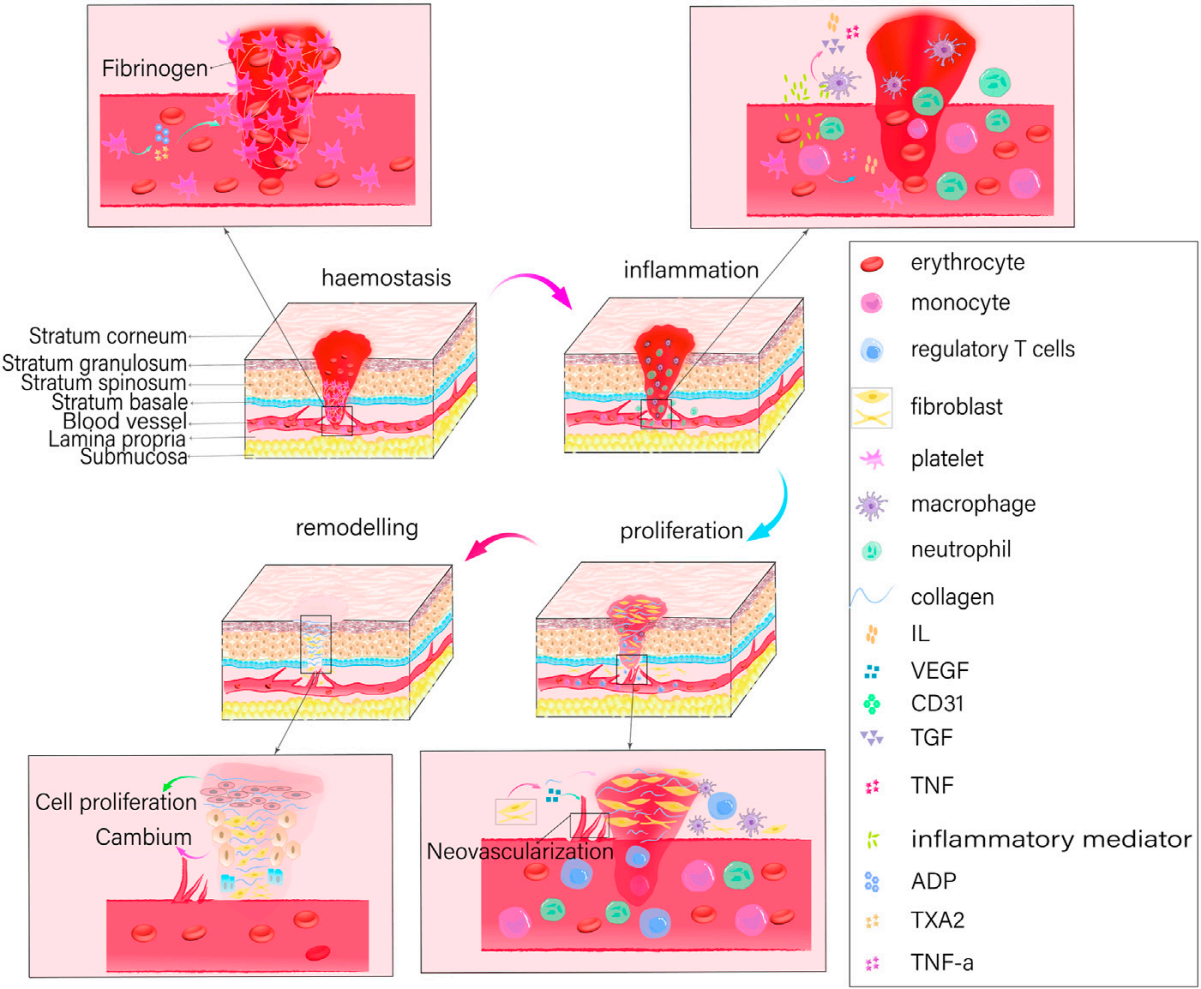
The process of wound healing*.

The healing process for oral and maxillofacial wounds is identical to that of skin wounds and involves four distinct phases: hemostasis, inflammation, proliferation, and remodeling (Figure 1). To preserve its structural integrity, an organism initiates the wound healing process by first establishing hemostasis. It then induces key cells involved in healing, such as neutrophils, monocytes, endothelial cells, and fibroblasts, to interact with signaling molecules that regulate cell synthesis. In addition, it eliminates damaged or necrotic tissues and microorganisms, and stimulates the cells to release cytokines that promote the growth of new blood vessels (angiogenesis), the formation of granulation tissue, the deposition of collagen, and the re-growth of epithelial cells. Thus, the overall process facilitates rapid wound healing.

Owing to the high blood flow in the oral and maxillofacial regions, post-incision bleeding is observed to be more frequent and hematoma formation is facilitated. At the same time, strong tissue regeneration and repair capabilities are also demonstrated allowing the rapid healing of wounds. Nonetheless, the oral cavity mucosa is a multimicrobial habitat, and the wound is surrounded by a moist and bacterial environment due to the presence of salivary glands. In addition, facial wounds bleed more profusely, and direct exposure increases the risk of infection and scarring. Tissue engineering for oral and maxillofacial wound healing has thus far encountered the difficulty of developing adhesive tissues that can withstand humid environments and combat infections. Healing oral and maxillofacial wounds can be challenging due to various factors. The oral cavity serves as the entryway to the respiratory and digestive systems; actions such as coughing, sneezing, chewing, and swallowing can, therefore, result in mechanical interference with the wound. This interference may impede the proper fixation of the wound dressing, consequently hindering the process of wound healing. Additionally, oral movements involving the tongue and teeth may undermine the protective properties of the wound dressing, potentially resulting in its displacement and subsequent wound exposure (Wu et al., 2022). Moreover, the oral cavity is a dynamic, unstable, and moist environment that is constantly flushed with saliva and exposed to exogenous food stimuli. Sustaining attachment and retention of wound dressings is complicated by these factors, which can impede the healing of oral and maxillofacial wounds (Sun et al., 2023). When contrasting the oral mucosa and the skin, it is important to note that the functionalities of every stratum of the oral mucosa are analogous to those of the skin layers. However, the oral mucosa is equipped with salivary glands and is situated in a more moist, friction-prone, and bacterial-rich environment (Glim et al., 2013). Thus, in the context of restoring traumatic injuries to the oral and maxillofacial regions, it is imperative to develop dressings and medications that exhibit favorable adhesion, in addition to possessing effective antimicrobial and anti-inflammatory properties that can operate efficiently in humid environments.

**FIGURE 2:**
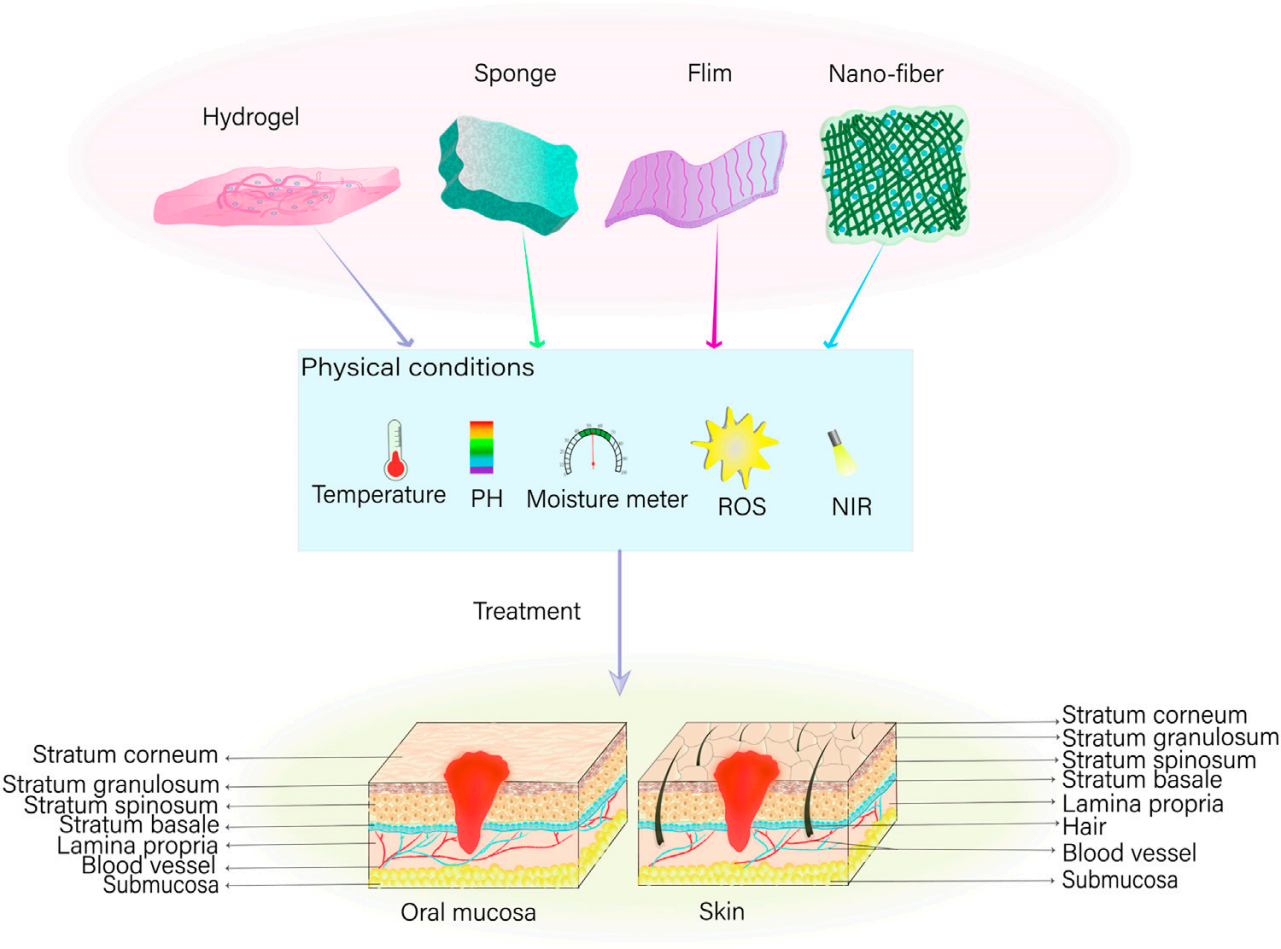
Tissue engineering strategies for wound healing*. In instances of oral mucous membrane or skin trauma, various dressings—including hydrogel, sponge, film, and nano-fiber—can be employed to precisely repair the affected area. This is feasible under suitable physical conditions encompassing temperature, potential of hydrogen (pH), humidity, and the concentration of reactive oxygen species (ROS), in addition to the implementation of near infrared (NIR) light-assisted irradiation on the wound.

The present article provides a comprehensive overview of tissue engineering strategies designed to promote wound healing, including the underlying mechanisms that facilitate the healing process. In particular, this review explores hydrogels, films, nanofibers, and foam sponges (Figure 2). Hydrogels can be described as shaped polymer network systems that resemble the extracellular matrix in nature (Zhang et al., 2015; Raina et al., 2022; Liu et al., 2023). These scaffolds function as three-dimensional porous networks and absorb surplus tissue exudate from the site of injury, thereby maintaining a moist wound environment that inhibits the proliferation of malignant tissues (Zhao et al., 2017). In addition, they allow oxygen to penetrate and cool the wound surface, thus alleviating patients’ discomfort and pain. By providing a physical barrier and expediting the healing process of wounds, hydrogels can function as a scaffold or temporary replacement for cell proliferation (Tomar et al., 2023). On the other hand, nanofibers have been reported to harbor anti-infective properties; thus, when loaded with chitosan, poly (ethylene oxide), and VEGF they can regulate the inflammatory response in the wound area more effectively (Xie et al., 2013). Furthermore, nanofibers can promote *in vitro* hemostasis and inhibit bacterial growth to prevent wound infections. Additionally, nanofibers can serve as a carrier for drug delivery, enabling the sustained release of drugs to locally act in the wound (Liu et al., 2017). Conversely, films effectively function as semi-permeable dressings and barriers to shield the trauma site from the effects of the external environment due to their thinness and elasticity. Furthermore, films offer a high degree of permeability to oxygen and water while simultaneously limiting the passage of bacteria, thus, providing a cellular barrier for wound healing (Liang et al., 2022). However, films lack a strong absorption capacity, thus rendering them suitable for mildly exuding wounds only (Liang et al., 2022). Foams, on the other hand, are comprised of polyurethane or silicone-based materials and facilitate a high degree of absorption and insulation. Not only are foams semipermeable, functioning as cellular barrier-like films (Shi et al., 2020), they also operate on a porous structure, thus, facilitating the ingress of moderate wound exudates, offering great filling capacity, and commendable shape memory (Landsman et al., 2017). In contrast, the interconnected porous structure of sponges renders them capable of absorbing large amounts of wound exudate and offer thermal insulating properties that increase tissue growth via hydrophilic and cellular interactions (Feng et al., 2019).

## 2 Hydrogels

### 2.1 Polysaccharide-based hydrogels

Chitin, the most abundant animal polysaccharide, contains a high amount of hydroxyl functional groups. By synthesizing hydroxypropyl chitin and combining it with tannic acid (TA) and Fe^3+^, composite hydrogels have been fabricated previously (Mehrabani et al., 2018). The resulting hydrogel scaffolds, enriched with hydroxyl groups, enhance the hydrophilicity of the material, thereby allowing for better absorption of exudate from the wound area. In addition, hydrogels have been reported to facilitate the sustained release of tannic acid (TA) and Fe^3+^, which promotes cell proliferation and migration at the wound site (Ma et al., 2020). Furthermore, these hydrogels have been observed to aid in increasing the deposition of collagen, thereby accelerating the wound healing process. In hydrogels composed of chitin-derived chitosan (CS) (Song et al., 2019), the amino group on the surface of CS interacts electrostatically with the negative charges on the surface of platelets and erythrocytes. This interaction promotes erythrocyte aggregation and activates platelets to release PDGF and TGF, thereby enhancing platelet aggregation and inducing the hemostatic process (Shen et al., 2006). At the same time, free amino groups engage with bacteria possessing negatively charged surfaces, resulting in the disintegration and rupture of the bacterial cell wall membrane. This process eradicates the bacteria by causing the leakage of the cell contents (Sahariah et al., 2014; Raina et al., 2022). In addition, snail glycosaminoglycan (the *A. fulica* glycosaminoglycan, AFG) and gelatin methacrylate (GelMA)-bonded dual-network hydrogels, which is inspired by the structure of snail mucus, releases AFG to reduce the production of inflammatory substances by activating the TLR4/NF-κB signaling pathway. Furthermore, it enhances the anti-inflammatory properties of macrophages by promoting their polarization towards the M2 type. The use of such hydrogels in the repair of wounds has been observed to significantly influence the early stage of wound healing, thereby increasing collagen deposition and neovascularization for rapid recovery of the wounded area (Zhou et al., 2023). Table 1.

**TABLE 1:** Sources of various polysaccharide hydrogels, their advantages, and their application in oral and maxillofacial wound healing.

Polysaccharide-based hydrogel	Sources	Advantages	Applications in oral and maxillofacial	Reference
Chitin/hydroxypropyl chitin	Invertebrates, crustacean shells, insect cuticles	By increasing the number of hydroxyl functional groups, the hydrogel scaffold can enhance its hydrophilicity. Additionally, it exhibits excellent biodegradability, preventing further harm to the wound when changing the dressing	The dressing’s high concentration of hydroxyl functional groups makes it especially effective in absorbing exudate at the wound site, which is particularly advantageous in the blood-rich oral and maxillofacial environment. Chitin hydrogels have the potential to eliminate the necessity for frequent dressing changes and minimize physical harm in the distinct context of oral wounds	Mehrabani et al. (2018), Khajavian et al. (2022)
Chitosan	A chitin-derived biopolymer	Chitosan enhances the clumping together of red blood cells and platelets and triggers the blood clotting process. The hydrogel’s surface area is enhanced to enhance its bio-adhesion, while its antibacterial properties are achieved through interaction with negatively charged bacteria	Chitosan aids in the adherence of the dressing to moist tissues in the mouth and face, and decreases the likelihood of infection in wounds in the mouth. It has applications in treating periodontal disease, performing oral and maxillofacial surgery, and managing diseases affecting the oral mucosa	Shen et al. (2006), Gao et al. (2022), Yang et al. (2022)
Glycosaminoglycans	All mammalian cells	It reduces the production of inflammatory substances and encourages the differentiation of macrophages, while also enhancing the accumulation of collagen	The functional groups present in glycosaminoglycans establish hydrogen and ionic bonds with the functional groups found in the tissue. This interaction enhances the adhesion of the dressing to the tissue surface, thereby facilitating the attachment of moist oral and maxillofacial wounds	Morla (2019), Zhou et al. (2023)
Arabic gum	A dried exudation obtained from the stems and branches of acacia (fam. Leguminosae)	The hydrogel possesses exceptional emulsifying and film-forming characteristics, along with a notable capacity for swelling, which facilitates the delivery of drugs	The solubility of Arabic gum enables the efficient administration of medications and the accurate treatment of oral and maxillofacial wounds. The Arabic gum hydrogel has the ability to quickly soak up blood in bleeding oral and maxillofacial wounds, facilitating accelerated clotting process in blood-rich wounds	Singh et al. (2017), Soubhagya et al. (2020), Ahmadian et al. (2023)
Alginate	Brown seaweed	It combines with the fluid that comes out of a wound to create a solid structure that shields the wound and creates a damp environment for healing	The strong attraction of alginate to harmful microorganisms aids in the prevention of wound infection in the complex environment of the oral and maxillofacial region	Lee and Mooney (2012), Ahmad Raus et al. (2021)
Agar	Certain marine red algae	The hydrogel absorbs exudate from the wound area and creates a moist environment to facilitate healing	Agar hydrogel has the ability to enhance blood flow in the oral and maxillofacial area, efficiently absorbing excessive fluid from wounds and creating a damp environment that aids in the healing process	Belattmania et al. (2021), Cheng et al. (2022)
Bacterial Cellulose	Several bacteria, such as *Gluconacetobacter xylinus*	The material demonstrates favorable air and water permeability as a result of its cross-linking network structure, which facilitates the transportation of drugs	The bacterial cellulose’s microfiber network efficiently transports drugs to the wound site, while its excellent air and water permeability ensures structural stability in a humid environment. This can be utilized for tissue adhesion in a dynamic and moist oral environment, which is favorable for repairing wounds in the oral and maxillofacial regions	Gupta et al. (2020), Lohrasbi et al. (2020), Singh et al. (2021)
Xanthan gum	*Xanthomonas campestris*	The hydrogel contains a significant amount of hydroxyl and carboxyl groups, resulting in a high level of polarity and hydrophilicity. Consequently, it exhibits a significant swelling rate, which is advantageous for absorbing wound exudate. The carboxyl and hydroxyl groups on the tissue surface form interactions with the amine groups, thereby facilitating tissue adhesion	The functional groups of xanthan gum exhibit a significant rate of swelling, facilitating the absorption of exudate from the wound and enhancing the adhesion of the dressing to the tissue by interacting with the functional groups of the tissue	Nsengiyumva and Alexandridis (2022), Tang et al. (2022)

Among plant polysaccharides, Arabic gum (AG) polysaccharide is known for its exceptional emulsification and film-forming properties (Soubhagya et al., 2020). Similarly, pectin (PEC) is recognized for its remarkable adhesive properties to mucous membranes and its ability to reduce inflammation (Soubhagya et al., 2020). Accordingly, AG and PEC have been used as raw materials, while Naringin was incorporated to fabricate hydrogels (Alsakhawy et al., 2022). The AG-based hydrogel utilizes the tissue adhesion of PEC to release Naringin in a sustainable manner within the trauma region. This process effectively reduces the expression of inflammatory factors and controls the inflammatory response. Additionally, the hydrogel scavenges free radicals and prevents lipid peroxidation due to its antioxidant properties (Cavia-Saiz et al., 2010). It also enhances cell proliferation and differentiation by up-regulating the expression level of TGF-β mRNA. Furthermore, the hydrogel induces wound contraction, leading to faster healing, and promotes the expression of vascular endothelial growth factor, which stimulates neovascularization (Santos et al., 2021; Alsakhawy et al., 2022). This increased blood supply provides nutrients to the tissues at the trauma site and facilitates tissue regeneration.

Alginate anionic polysaccharides, which are obtained from algae, produce a gel matrix capable of protecting wounds and averting infection by interacting with cations in wound exudates and dissolving a fraction of them (Lee and Mooney, 2012). In addition, they have the potential to facilitate wound healing by creating a moist environment (Ahmad Raus et al., 2021). Accordingly, hydrogels composed of sodium alginate (SA) and gelatin have been reported to undergo a reduction in their equilibrium solubility, preventing re-injury caused by physical pressure on the wound at the time of application due to swelling (Aderibigbe and Buyana, 2018; Deng et al., 2022). Furthermore, SA adheres to tissues via the oxidation of functional groups of the molecular chain, which results in the formation of imines (Han et al., 2017; Reakasame and Boccaccini, 2018; Gao et al., 2019). Moreover, Agar polysaccharides (AGAR), which are extracted from seaweed (Belattmania et al., 2021), have been infused with fumaric acid (FA) to modify and synthesize silver nanoparticles (Ag NPs) resulting in the development of AA-Ag-FA hydrogels (Basha et al., 2020). FA promotes the angiogenic pathway to stimulate neovascularization, increase granulation tissue formation, and remodel collagen, thus accelerating wound healing. In addition, the fabrication of AGAR@PVA-TA hydrogels by mixing Agar and polyvinyl alcohol (PVA), followed by the subsequent introduction of tannic acid (TA) have been reported (Cheng et al., 2022). The use of TA improves the mechanical strength of the hydrogel owing to hydrogen bonding between the phenolic hydroxyl group of TA and the hydroxyl groups of Agar and PVA. At the same time, it enhances the ability of the hydrogel to capture erythrocytes while absorbing bleeding from the wound and promotes the production of thrombin, which expedites the hemostatic process (Ge et al., 2019; He et al., 2020; Cheng et al., 2022).

In addition to the above, bacterial cellulose (BC), derived from microorganisms, can be transformed into a hydrogel that can be loaded with drugs and specifically delivered to wound sites, promoting the healing process (Gupta et al., 2020; Lohrasbi et al., 2020). The addition of collagen to the BC-based hydrogel creates a porous structure with a larger pore size, which allows the cellulose nano fibers (CNFs) to intertwine with collagen, forming a denser network of fibers. This promotes the inward proliferation and growth of fibroblasts, as observed in studies by Moraes et al. (2016) and Pasaribu et al. (2023) (Moraes et al., 2016; Pasaribu et al., 2023). In addition, Chen et al. (2023) added Glucan and Xylan to BC hydrogel in order to enhance its ductility, improve tissue adhesion, and promote wound healing (Chen et al., 2023). Xanthan gum (XG) comprises another polysaccharide obtained from microorganisms. By combining the XG network with a covalent polyacrylamide network, the fabrication of a porous and stable hydrogel through hydrogen bonding was reported (Tang et al., 2022). This interaction relied on the abundant carboxyl and hydroxyl groups of XG to non-covalently interact with the amine groups on the tissue surface, enabling adhesion with the tissues at the wound site.

### 2.2 Carrier hydrogels

**FIGURE 3:**
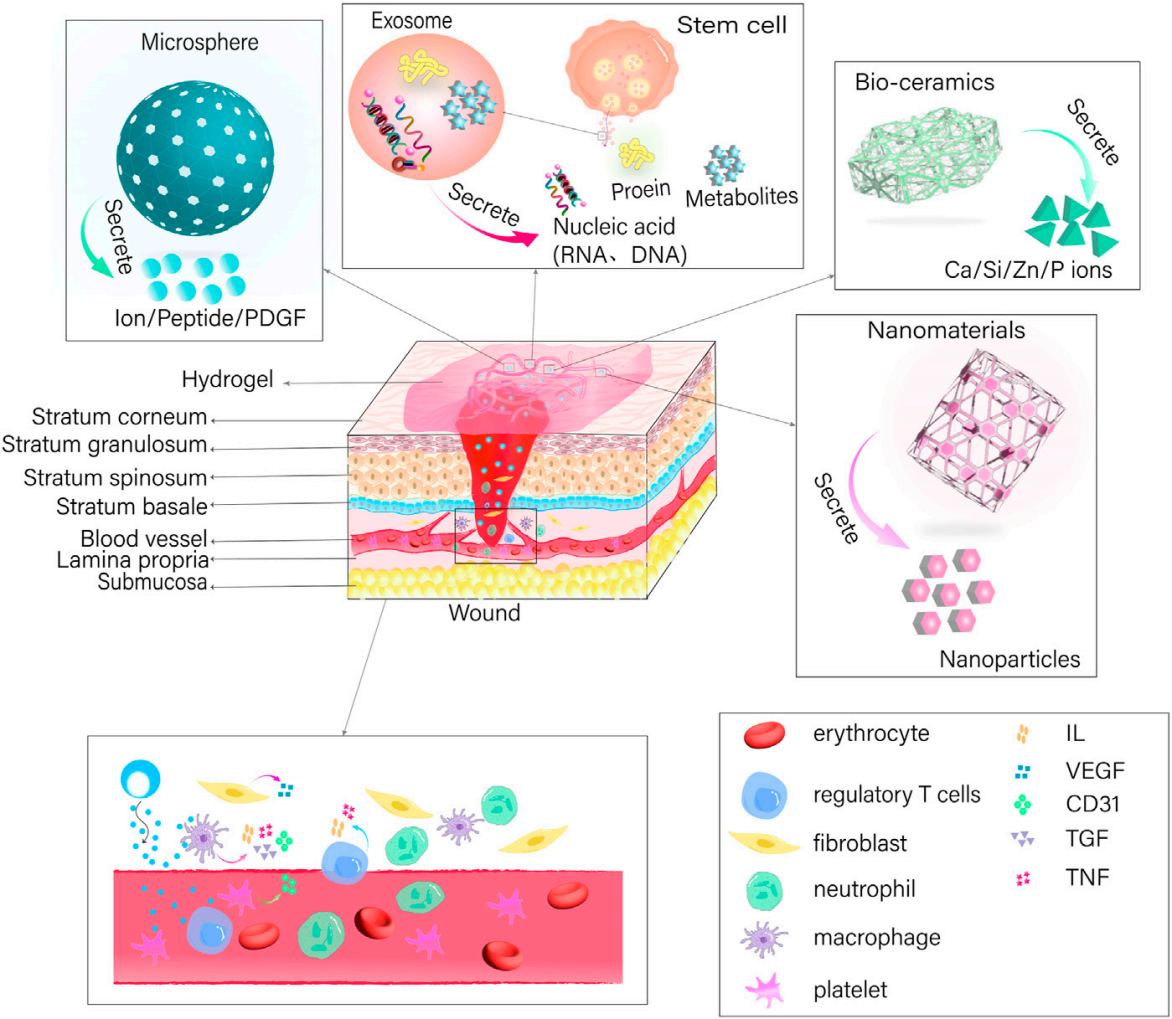
Hydrogels for precise wound healing*.

Nanomaterials possess the capacity to inflict damage upon bacteria by directly engaging with the bacterial cell wall membrane via their sharp edges (Akhavan and Ghaderi, 2010; Lu et al., 2022). Alternatively, they possess the capability to disturb cell membranes and provoke oxidative stress through the production of reactive oxygen species (ROS), interfering with the glycolysis process of bacteria, and disrupting bacterial DNA, among other antimicrobial effects (Kumar et al., 2011; Lakshmi Prasanna and Vijayaraghavan, 2015). Nanoparticles typically exert their antimicrobial effect through three mechanisms: the release of metal ions, the induction of oxidative stress, and non-oxidative mechanisms (Wang et al., 2017a; Barczikai et al., 2021). These mechanisms allow nanoparticles to enter bacterial membranes and interfere with the formation of bacterial biofilms. Controlled release of nanoparticles to specific locations can be achieved by utilizing the three-dimensional network structure of hydrogel (Gao et al., 2016). In this context, the synthesis of water-soluble polyethylene glycol (PEG) polymers containing catechol was achieved by imitating the adhesive properties of mussel proteins, which was then oxidized using silver nitrate to form a hydrogel (Fullenkamp et al., 2012). In addition, the incorporation of copper oxide nanoparticles (CuO NPs) (Ounkaew et al., 2020; Abdollahi et al., 2021) and silver nanoparticles (Ag NPs) (Yu et al., 2020), into carboxymethylated starch-based hydrogels have been reported. Furthermore, curcumin-cyclodextrin-characterized Ag NPs have also been incorporated into BC hydrogels (Gupta et al., 2020; Lyu et al., 2020) resulting in bactericidal action via release of nanoparticles that hinder the formation of biofilms and generate ROS. This disrupts the permeability of bacterial membranes, impairs bacterial proteins and DNA, and results in the leakage of cellular contents. Moreover, the nanoparticles incorporated into the hydrogel create a rough surface, which enhances the ability of cells to attach, multiply, and move, ultimately promoting the healing of wounds. Figure 3; Table 2.

**TABLE 2:** Types of nanoparticles loaded in various hydrogels, as well as their significance and applications in the oral and maxillofacial wound healing.

Carrier hydrogel	Nanoparticle type	Meanings	Applications in oral and maxillofacial	Reference
Nanoparticles hydrogel	Metal nanoparticles	Nanoparticles have the ability to hinder the formation of bacterial biofilms through different mechanisms, such as the discharge of metal ions, oxidative stress, and non-oxidative mechanisms. They have the ability to infiltrate bacterial membranes, causing harm to bacterial proteins and DNA, and producing antimicrobial outcomes	Metallic nanoparticles possess antimicrobial properties, which have been proven to eliminate bacteria and promote the healing of wounds in the intricate oral and maxillofacial environment. Additionally, it can be employed for dental implants and the treatment of periodontal disease	Wang et al. (2017a), Barczikai et al. (2021)
Exosome hydrogel	Stem cell exosome	Downregulating the expression of inflammatory mediators modulates the inflammatory reaction, promotes the formation of new blood vessels, improves the deposition of collagen, and controls the ratio to minimize the formation of scars	Because of the distinctive positioning of the oral and maxillofacial area, exosomes can be accurately transported to the site of the wound. They have a vital function in controlling inflammation, stimulating cell growth, and exerting a positive effect on tumor treatment, periodontal regeneration, and wound healing. Exosomes play a substantial role in the repair and regeneration of oral and maxillofacial nerves	Peng et al. (2020), An et al. (2021), Yang et al. (2021), Ma et al. (2022), Zhou et al. (2022), Song et al. (2023)
Hydrogels loaded with bio-ceramics	Silicate bio-ceramics/bioactive hard stone/bioactive glass	The process involves increasing the production of keratin and epidermal growth factor, which stimulates the growth and specialization of skin cells. It also releases calcium, silicon, and phosphorus ions, which prevent the growth of bacteria. Additionally, it promotes the production of vascular endothelial growth factor, which encourages the formation of new blood vessels	Biocompatible glass can be used to regenerate both hard and soft tissues. It releases calcium ions that generate an alkaline environment, promoting wound healing in areas with acidic or neutral pH levels. Furthermore, it engages with adjacent tissues to create imine bonds, thereby increasing adhesion. Additionally, silica ions play a role in the process of neovascularization	Zeng et al. (2016), Gao et al. (2019)
Microsphere hydrogel	Gel microspheres/copolymer microspheres/biopeptide microspheres	Microspheres possess a diverse array of bio-functionalities. These substances have the ability to enter the skin and release their contents in a targeted and regulated manner. Additionally, they have the ability to suppress inflammatory reactions, enhance the growth and movement of cells, promote the formation of new blood vessels, and stimulate the accumulation of collagen	The distinctive architecture of the microspheres enables precise administration of medication to address conditions such as periodontal disease, oral implant restoration, and oral infections	Moioli et al. (2007), Hakim et al. (2021), Ren et al. (2021), Ming et al. (2024)

The integration of exosomes into a hydrogel offers a means to transport and maintain exosomes by leveraging their three-dimensional architecture and compatibility with living organisms (Liu et al., 2017). Some examples of this include the use of adipose-derived mesenchymal stem cell exosome (ADSCs-EXO) loaded into Pluronic F-127 hydrogel (Zhou et al., 2022). These exosomes are enriched with miRNAs, which have the ability to regulate inflammatory responses by reducing the expression of TNF-a and IL-6 (Zhou et al., 2022). Additionally, they activate the PTEN/PI3K/AKT signaling pathway (Ma et al., 2022) and increase the expression of CD31 levels (Zhou et al., 2022) in order to promote neovascularization. In addition, the uptake and internalization of ADSCs-EXOs by fibroblasts can lead to an increase in the expression of N-cadherin, cyclin-1, and PCNA (Hu et al., 2016). This effect is achieved through the activation of the PI3K/AKT pathway and the Wnt/β-catenin pathway (An et al., 2021; Ma et al., 2022; Song et al., 2023), as well as the high expression of a-SMA (Zhou et al., 2022). These mechanisms ultimately promote fibroblast proliferation and migration. Activation of the TGF-β/Smad signaling pathway, as demonstrated by Liu et al. (2022) (Liu et al., 2022)promotes the production of collagen, which in turn enhances the process of re-epithelialization and accelerates wound healing. Regulating the proportion of type I and type III collagen and increasing the ratio of TGF-β3 to TGF-β1 during the later stages of wound healing has the potential to facilitate the remodeling of the extracellular matrix and minimize scar formation (Wang et al., 2017b). In addition, exosomes derived from mesenchymal stem cells (MSCs) present in human umbilical cord blood has the capacity to enhance cell proliferation for wound healing and suppress scar formation by inhibiting the differentiation of myofibroblasts (Zhang et al., 2021). This is achieved through the suppression of the TGF-β1 signaling pathway-induced overexpression of a-SMA and type I collagen (Zhang et al., 2021).

Silicate bio-ceramics have the ability to upregulate keratin, integrin β1, and epidermal growth factor, which in turn stimulates the proliferation and differentiation of epidermal cells (Wang et al., 2020; Fan et al., 2022). This effect is achieved by regulating the Wnt/β-catenin and ERK signaling pathways. One of these materials, known as bioactive hard stone (HS), facilitates the gradual release of Ca, Si, and Zn (Li et al., 2017). Another type of bio-ceramic material is bioactive glass (BG), which has the ability to release Ca, Si, and P (Wang and Tang, 2022). The hydrogel synthesis involves combining BG coated with gelatin, CS, and PDA with silver nanoparticles that are capped with curcumin (Cur-Ag NPs) (Tomar et al., 2023). The combined effect of BG and Cur-Ag NPs promotes the growth and movement of fibroblasts towards the wound site, leading to increased levels of VEGF, COL1a, and TGF-β (Tomar et al., 2023). This, in turn, enhances angiogenesis and accelerates the wound healing process. In addition, the incorporation of HS or BG into an SA-based hydrogel (Lansdown et al., 2007; Brauer, 2015; Gao et al., 2019), can lead to the release of ionic cross-linking of SA, resulting in the formation of a porous network scaffold (Lansdown et al., 2007; Brauer, 2015; Gao et al., 2019). This inhibits bacterial growth, while also promoting the multiplication and movement of keratinocytes in the area of the wound and increasing angiogenesis and re-epithelialization through the enhancement of intracellular signaling pathways. In addition, the use of lithium calcium silicate bioactive ceramic wound dressings has been shown to impact the inflammatory response in wound healing by promoting the polarization of macrophages towards the anti-inflammatory M2 type (Huang et al., 2018; Waasdorp et al., 2021; Fan et al., 2022). This is achieved by reducing the expression of IL-1β, IL-6, and TNF-a, while increasing the expression of IL-10. For example, BG has the ability to release Si ions, which can stimulate the increased expression of VEGF and bFGF. This, in turn, promotes the growth of vascular endothelial cells and fibroblasts in the wound area and enhances angiogenesis, thus expediting wound healing (Yu et al., 2016; Li et al., 2017; Zhou et al., 2018; Gao et al., 2019).

In addition, hydrogels have been employed as carriers to encapsulate pro-healing substances in the form of microspheres for the purpose of wound repair. For instance, a manganese-doped dopamine-derived hollow carbon sphere (MnO_x_/HNCS) were synthesized using dopamine and manganese acetylacetonate in combination with SiO_2_ microspheres as templates, which is cellulose-based gel microspheres (Lu et al., 2022). The gel microspheres promoted wound healing by suppressing inflammatory reactions and inducing the expression of healing-associated protein factors through the use of photothermal effects and the release of Mn^2+^ ions. In addition, they enhanced the growth, attachment, and movement of keratinocytes and fibroblasts (Tenaud et al., 1999; Lu et al., 2022), which in turn promoted the accumulation of collagen and facilitated the process of re-epithelialization, thereby expediting wound healing. Moreover, platelet-derived growth factor receptor (PDGF) has also been embedded in polylactic acid-glycolic acid copolymer (PLGA) (Xu et al., 2020). These microspheres were then combined with a solution containing CS/β-GF to create a PDGF-PLGA hydrogel. The PDGF was dispersed within the porous structure of the PLGA microspheres and subsequently released into the wound site via the characteristics of the CS hydrogel. This release aimed to stimulate the growth of fibroblasts and endothelial cells, thereby promoting the development of granulation tissue and neovascularization, resulting in wound healing. The production of CS-C/OPM/β-GP hydrogel has also been demonstrated by incorporating functionalized catechol chitosan (CS-C) into integral active oyster peptide microspheres (OPM) and sodium β-glycerophosphate (β-GP) (Zhang et al., 2021; Raina et al., 2022). The highly compact and consistent surface of the OPM facilitated the gradual release of oyster peptide, effectively reducing inflammatory responses by decreasing the expression of TNF-a and IL-6. In addition, it promoted the formation of new blood vessels by increasing the expression of VEGF and Ki-67, as well as the deposition of collagen fibers, which are crucial for wound healing (Zhang et al., 2021).

## 3 Nanofibers

### 3.1 Inorganic nanofibers

Carbon nanotubes (CNTs) have been employed as frameworks for precise drug delivery by binding with hydrophilic CS, enveloping CS around carbon nanotubes to augment the adhesive characteristics. The hydrophilic part of CS is exposed to the surroundings in order to facilitate the absorption of exudate from the wound area and maintain a moist microenvironment (Kittana et al., 2018). This moist environment stimulates the growth and movement of fibroblasts, increases collagen deposition, and aids in re-epithelialization.

By leveraging the structure of the nanofiber to imitate the extracellular matrix, CNTs were employed to improve the scaffold’s mechanical strength and structure, enhance adhesion between the biomaterial scaffold and tissue cells, and stimulate cell proliferation (Eivazzadeh-Keihan et al., 2019). By means of interaction with bacteria, disruption of bacterial cell membranes, and subsequent leakage of cellular contents, the integration of CNTS into the fibrous structural network of BC impedes cell proliferation and restricts activity, thereby exerting an antimicrobial effect (Kang et al., 2008; Akasaka and Watari, 2009). Furthermore, by facilitating cell adhesion and proliferation to form granulation and ensuring air-liquid exchange at the wound site via its porous structure, CNTs promote wound closure and re-epithelialization. Additionally, by stimulating neovascularization and increasing capillary density to advance tissue vascularization, BC and CNTs promote high VEGF expression in a synergistic manner, thereby accelerating the wound healing process (Khalid et al., 2022).

### 3.2 Organic nanofibers

Biopolymeric nanofibers synthesized via electrostatic spinning demonstrate excellent biocompatibility and biodegradability, and demonstrate significant potential for use as scaffolds when doped with drugs to stimulate cellular adhesion and proliferation (Farhaj et al., 2023). This can enhance the process of tissue regeneration and repair (Alavarse et al., 2017). By incorporating polysaccharide-based materials into nanofiber scaffolds, the healing process of wounds can be enhanced. This is achieved by delivering functional polysaccharide biopolymers directly to the wound site, controlling inflammatory reactions, promoting the growth and movement of fibroblasts, and increasing the deposition of collagen (Iacob et al., 2020).

Hyaluronic acid (HA) nanofibers consist of glucosamine, glucuronic acid, and functional groups including polyhydroxy and polycarboxylate surface groups. These groups have the ability to interact with receptors on the cell surface in order to promote the movement and proliferation of fibroblasts and endothelial cells towards the site of the wound (Li et al., 2018; Debele and Su, 2022). In order to control the bleeding, HA absorbs the blood at the site of the wound via the nanofibers’ structure and acts as an adhesive framework for platelets to promote coagulation (Astaneh and Fereydouni, 2023). Furthermore, HA stimulates the polarization of macrophages towards the M2 subtype and inhibits the release of growth factors from the cells, thereby promoting fibroblast and endothelial cell proliferation and facilitating granulation and re-epithelialization.

Chitosan nanofibers, eucalyptus oil, and cellulose acetate nanofibers have also been used to prepare wound dressings (Elbhnsawi et al., 2023). In this context, the arrangement of fibers is crucial in attracting bacterial cells and ensuring their adhesion to the nanofiber surface; on the other hand, the inclusion of 1,8-eucalyptin in eucalyptus oil enhances the permeability of bacterial cytoplasmic membranes (Aldoghaim et al., 2018). Subsequently, the disruption of the cell membranes results in the lysis of bacterial cells and the release of cellular contents, ultimately resulting in bactericidal action. In addition, the dressing stimulates the development of granulation tissue and the deposition of collagen, as well as the growth of fibroblasts, endothelial cells, and keratinocytes. This is achieved by increasing the levels of mRNA expression of TGF-β1, type I and type III collagen, which in turn leads to the formation of new blood vessels and the promotion of the regrowth of epithelial cells, facilitating the healing of wounds.

## 4 Biofilms

### 4.1 Silk fiber membranes

Silk fiber membranes (SF membranes) enhance cell migration and proliferation and facilitate wound healing by activating the NF-jB signaling pathway, which is characteristic of the wound healing process, both *in vitro* and *in vivo*, thereby inducing the expression of multiple growth factors (Li et al., 2022). In this context, SF membranes created by enclosing surface aminated liposomes (NH_2_-LIPs) containing leptin have been reported previously, which were subsequently submerged in a polydopamine (PDA) solution (Qian et al., 2020; Schäfer et al., 2022). The NH_2_-LIPs were then grafted onto the surface of the SF membranes through a reaction between the amino group and the catechol moiety of PDA. The application of PDA increases the water-attracting properties of the fibers and improves the ability of fibroblasts to stick to the fiber membranes. In contrast, the NH_2_-LIPs inhibits the degradation of leptin bioactivity caused by the organic solvent and oral enzymes, while also quickly releasing leptin into the damaged tissues. Moreover, it induces a notable upregulation of angiogenic factors via pathways like STAT3 and MAPK23, and enhances the growth and formation of blood vessels by promoting the proliferation of endothelial cells, thus facilitating the regeneration of oral mucosal tissue.

Moreover, flat silk cocoons (FSCs) have been reported to be able to generate a novel form of natural SF membrane that can be used directly without the requirement of a lysis and regeneration procedure (Li et al., 2022). This membrane exhibits controllable dimensions and possesses a porous layer structure, in addition to displaying exceptional mechanical properties. FSC has the ability to attract fibroblasts to the dermis and stimulate the production of growth factors, collagen, and other components of the extracellular matrix. This process enhances the speed at which damaged tissues can be repaired.

### 4.2 Collagen base

Collagen, a prominent constituent of the extracellular matrix, possesses the capacity to create intricate networks and merge together. It serves as both a carrier for drug delivery and a scaffold for cell growth by cross-linking and self-aggregating to create durable fibers that enhance tissue repair or regeneration at the wound site. Accordingly, collagen films serve as barriers at the wound site to prevent infection, while also gradually releasing the drug into the wound (Chattopadhyay and Raines, 2014). The biodegradable collagen film acts as a scaffold for fibroblasts, promoting cell growth and collagen synthesis. In addition, the degradation products promote the growth of fibroblasts and vascular endothelial cells, as well as facilitate the movement of keratinocytes. These cells generate and release a range of growth factors to enhance the development of extracellular matrix, angiogenesis, and re-epithelialization of tissues.

### 4.3 Covalent organic framework films

metal organic frameworks (MOFs) are categorized as porous polymeric substances with antimicrobial, tissue regeneration-enhancing, and drug-carrying properties (Chen et al., 2018). In the context of wound healing, Peng et al. (2022) (Peng et al., 2022) developed a bimetallic organic frameworks (BMOFs)-based dressing (BMOF-DMR) using Cu/Zn-rich metal organic frameworks (MOFs) and glucose oxidase (GOx). However, zeolitic imidazolate framework-8 (ZIF-8) comprises the most extensively utilized MOF for wound repair. In acidic wound conditions, the discharge of Zn^2+^ stimulates the production of ROS within the bacterial cells, resulting in the onset of oxidative stress, which subsequently disrupts the synthesis of the disorganization of bacterial cell genes, and ultimately leading to the death of the bacteria. In addition, the introduction of Cu^2+^ into the BMOF-DMR and the continuous release of Cu^2+^ stimulates the expression of VEGF and CD34, which facilitates angiogenesis, accelerates wound healing, and facilitates tissue regeneration.

Covalent organic frameworks (COFs), which may take the form of porous crystalline polymers, also serve as drug delivery vehicles due to their exceptional biocompatibility and high porosity (Zou et al., 2022; Bukhari et al., 2023). The formation of Cur @COF/PCL NFM wound dressings has been documented to occur when Cur is loaded with COFs and doped into polycaprolactone (PCL) nanofiber films via electrostatic spinning (Zou et al., 2022; Mohajer et al., 2023). Cur is released in response to PH at the wound site and interferes with Fts Z, an essential protein for bacterial cell division (Kaur et al., 2010), which inhibits bacterial proliferation. Concurrently, it promotes rapid wound healing by assisting in the reduction of TNF-a levels, which in turn decrease the concentration of inflammatory cells at the site of the wound, and by increasing VEGF expression, which stimulates angiogenesis and enhances fibroblast proliferation and migration into the wound.

By utilizing cyclodextrin metal organic framework (CD-MOF) as a template, the synthesis of a porous fiber platform containing ultrafine silver nanoparticles was also facilitated in an earlier study. Meanwhile, in a study conducted by He et al. (2023) (He et al., 2023) Ag @MOF @PDA dressings were produced by encapsulating PDA on the surface of the framework. By enhancing the water stability of the scaffolds through the synergistic action of PDA on the surface and internal cyclodextrins, CD-MOF becomes less susceptible to collapse. This, in turn, facilitates the sustained release of Ag^+^, which is essential for sustaining the long-term effective concentration. Furthermore, when near-infrared light irradiation is applied to the wound site, the adhesion between PDA and bacteria initially binds to the bacteria at the wound site. The disruption of the bacterial cell wall membrane by the subsequent release of a substantial quantity of Ag^+^ causes the cell contents to escape and the bacteria to perish.

## 5 Foam/sponge dressings

Polyurethane foam (PUF) has a high capacity to absorb, along with strong mechanical stability and flexibility (Sahraro et al., 2016). This makes it effective in absorbing excessive exudate at the wound site and promoting tissue growth. In addition, lignin-based polyurethane foams (LPUFs) as antimicrobial PUF dressings can be acquired by incorporating Ag NPs that are capped with lignin (Morena et al., 2020; Li et al., 2022). The phenolic hydroxyl groups in lignin interfere with the movement of protons across the cell membrane by reducing the pH within the membrane (Li et al., 2022). This leads to the breakdown and rupture of the cell membrane, causing the release of cellular contents. In addition, Ag NPs adhere to the cell membrane via electrostatic interactions, releasing Ag^+^ ions that interact with bacterial cell proteins and DNA residues, resulting in antimicrobial effects. The imidazole bromide was synthesized by combining a mixture of bromoethanol and imidazole in a solution of acetonitrile. The mixture obtained was employed to generate cationic polyurethane foam at room temperature (Ding et al., 2019). Moreover, the imidazole cation interacts electrostatically with the negative charge of the bacterial cell wall, leading to a decrease in the production of inflammatory cells in the wound area. Additionally, it regulates the expression levels of TNF-a, IL-1, and IL-6 to control the inflammatory response.

Earlier studies also report the use of sericin to synthesize Ag NPs, which were then combined with curcumin (Cur) to create an antimicrobial agent: sericin-Ag NPs/Cur (Se-Ag/Cur) (Jiang et al., 2023). The antimicrobial agent was enclosed within SA-CS (SC) and immersed in a CaCl_2_ solution to create a composite sponge called SC/Se-Ag/Cur. Sericin exhibits favorable biocompatibility, minimal immunogenicity, and promotes cell adhesion (Guo et al., 2023). These properties enhance the hydrophilicity and solubility of the composite sponge, facilitating the absorption of wound exudate. In addition, it forms a three-dimensional interconnected porous structure by utilizing the electrostatic interactions between SA and CS, as well as the ionic interactions between SA and Ca^2+^. This structure effectively prevents dehydration and the accumulation of exudate during the wound healing process, while also promoting the delivery of nutrients. The composite sponge, formed by the combination of Ag NPs and Cur, effectively suppressed inflammation by decreasing the expression of TNF-a and enhanced the formation of new blood vessels by promoting early CD31 expression in the wound tissue (Jiang et al., 2023). Additionally, Cur facilitated the development of granulation tissue, tissue remodeling, and collagen formation (Table 3).

**TABLE 3:** Advantages of Nanofibers, Biofilms, and Foam Sponges for Oral and Maxillofacial wound healing.

Wound dressing	Advantages	Applications in oral and maxillofacial	Reference
Inorganic nanofiber	Inorganic nanofibers improve the mechanical robustness of drug delivery scaffolds	It has the potential to be utilized in oral regenerative medicine as a framework to transport medications to the site of the wound and promote the growth of cells. It is also used in oral implants for the purpose of guided bone regeneration and in the prevention of wound infections in oral infections	Eivazzadeh-Keihan et al. (2019), Castro-Rojas et al. (2021)
Organic nanofiber	Organic nanofibers possess the properties of being both biocompatible and biodegradable, which enhance the ability of tissue cells to adhere and facilitate the process of tissue repair	Nanofibers can be synthesized utilizing hyaluronic acid, chitosan, or cellulose for the purpose of absorbing exudate at oral and maxillofacial wounds and stimulating tissue cell proliferation to facilitate repair	Alavarse et al. (2017), Iacob et al. (2020)
Silk fiber membranes	The application of silk fiber film can improve the attachment of fibroblasts, accelerate their growth, induce tissue regeneration, and expedite the healing process of wounds	The regenerative effect of silk fiber films makes them suitable for treating periodontitis diseases by promoting the growth of oral mucosal cells	Serôdio et al. (2019), Qian et al. (2020), Schäfer et al. (2022)
Collagen base	Collagen membranes can function as scaffolds for the delivery of drugs and the growth of cells, while also acting as barriers to shield wounds from infection	Collagen membranes in maxillofacial wounds serve to prevent bacterial infection, while also promoting cell proliferation and reducing facial scarring through collagen degradation	Chattopadhyay and Raines (2014)
Covalent organic framework film	Covalent organic frameworks films are polymer materials with porosity that exhibit resistance to infection, promote the growth of cells, and facilitate the regeneration of tissues	The oral and maxillofacial region harbors a diverse range of microorganisms, rendering it vulnerable to infection after experiencing trauma. Covalent organic framework films have advantageous properties in preventing wound infections, safeguarding wounds, stimulating cell proliferation, and expediting wound healing	Chen et al. (2018), Peng et al. (2022)
Foam/Sponge dressing	Foam and sponges have a high capacity to absorb liquid because of their distinct porous structure, which allows them to efficiently soak up moderate quantities of fluid from wounds	Foams and sponges have exceptional hygroscopic and moisturizing characteristics that effectively absorb exudate during oral wound bleeding, thereby creating a conducive environment for wound healing. Furthermore, they function as frameworks for precise administration and discharge of drugs, facilitating the growth of tissue cells, thereby expediting the process of wound healing. This is especially crucial in the context of injuries to the mouth and face, as well as the process of healing surgical wounds	Landsman et al. (2017), Feng et al. (2019), D’Amico et al. (2023)

## 6 Conclusion and perspectives

Hydrogels have become an intense topic of interest in modern society, where difficulties and uncertainties permeate every aspect. By capitalizing on the exceptional water absorption and retention properties of the three-dimensional structure, exudate from the wound is absorbed and a moist environment is maintained; this simultaneously lowers the temperature of the wound site and promotes the healing process. Additionally, the porous structure of the hydrogel facilitates the ingress of oxygen and water, which promotes the proliferation and growth of tissue cells in the vicinity of the wound. Adhesion of wound dressings to tissues in the oral and maxillofacial region is significantly hindered by the moist environment of the mucosa in the oral cavity and the blood of superficial skin wounds. Therefore, by capitalizing on the hydrogel’s gentle viscoelasticity and the biomaterials’ functional groups incorporated in its synthesis, an exceptional adhesion with the tissues can be achieved. However, despite possessing commendable biocompatibility and biodegradability, hydrogels fail to exhibit satisfactory mechanical properties. Despite having a network structure, hydrogels are susceptible to the impact of physiological processes occurring in the oral environment, including mastication and swallowing. Furthermore, when employed to absorb exudate from wounds, hydrogels that consist of porous hydrophilic polymer networks comprised of polymers experience an increase in mass and volume due to the presence of a considerable number of hydrophilic groups in the polymer backbone. This expansion leads to a further degradation of the hydrogels mechanical properties and stability. Additionally, the swelling hydrogel obstructs and compresses the tissue adjacent to the site of the wound, thereby impeding the tissue’s ability to heal precisely (Wu et al., 2022). In light of the aforementioned drawbacks of hydrogel, it is expected that forthcoming research will explore the application of hydrogel for wound healing, which would possess enhanced mechanical properties, decreased vulnerability to external mechanical disturbances, and superior water absorption and non-expansion characteristics.

## Abbreviations

a-SMA, Alpha-smooth muscle; bFGF, Basic fibroblast growth factor; CD31, Platelet endothelial cell adhesion molecule-1, PECAM-1/CD31; CD34, Recombinant cluster of differentiation 34; Col1a, Collagen, type I, alpha 1; IL-6, Interleukin- 6; IL-10, Interleukin- 10; IL-1β, Interleukin- 1β; PCNA, Proliferating cell nuclear antigen; PDGF, Platelet-derived growth factor; TGF-β, Transforming growth factor-β; TNF-a, Tumor necrosis factor alpha; VEGF, Vascular endothelial growth factor.
